# An economic evaluation: Simulation of the cost-effectiveness and cost-utility of universal prevention strategies against osteoporosis-related fractures

**DOI:** 10.1002/jbmr.1758

**Published:** 2013-02

**Authors:** Léon Nshimyumukiza, Audrey Durand, Mathieu Gagnon, Xavier Douville, Suzanne Morin, Carmen Lindsay, Julie Duplantie, Christian Gagné, Sonia Jean, Yves Giguère, Sylvie Dodin, François Rousseau, Daniel Reinharz

**Affiliations:** 1 Département de médecine sociale et préventive, Faculté de Médecine, Université LavalQuébec, Québec, Canada; 2 Département de génie électrique, Faculté des Sciences et de génie, Université LavalQuébec, Québec, Canada; 3 Department of internal medicine, Faculty of Medicine, McGill UniversityMontreal, Quebec, Canada; 4 Centre de recherche du centre hospitalier universitaire de Québec (CRCHUQ), Faculté de Médecine, Université LavalQuébec, Québec, Canada; 5 Institut de santé publique du Québec (INSPQ)Québec, Québec, Canada; 6 Département de biologie moléculaire, biochimie médicale et pathologie, Université LavalQuébec, Québec, Canada; 7 Département d'obstétrique et gynécologie, Université LavalQuébec, Québec, Canada

**Keywords:** OSTEOPOROSIS, SCREENING, COMPUTER SIMULATIONS, COST-EFFECTIVENESS, COST-UTILITY, PREVENTION, BONE FRACTURES

## Abstract

A patient-level Markov decision model was used to simulate a virtual cohort of 500,000 women 40 years old and over, in relation to osteoporosis-related hip, clinical vertebral, and wrist bone fractures events. Sixteen different screening options of three main scenario groups were compared: (1) the status quo (no specific national prevention program); (2) a universal primary prevention program; and (3) a universal screening and treatment program based on the 10-year absolute risk of fracture. The outcomes measured were total directs costs from the perspective of the public health care system, number of fractures, and quality-adjusted life-years (QALYs). Results show that an option consisting of a program promoting physical activity and treatment if a fracture occurs is the most cost-effective (CE) (cost/fracture averted) alternative and also the only cost saving one, especially for women 40 to 64 years old. In women who are 65 years and over, bone mineral density (BMD)-based screening and treatment based on the 10-year absolute fracture risk calculated using a Canadian Association of Radiologists and Osteoporosis Canada (CAROC) tool is the best next alternative. In terms of cost-utility (CU), results were similar. For women less than 65 years old, a program promoting physical activity emerged as cost-saving but BMD-based screening with pharmacological treatment also emerged as an interesting alternative. In conclusion, a program promoting physical activity is the most CE and CU option for women 40 to 64 years old. BMD screening and pharmacological treatment might be considered a reasonable alternative for women 65 years old and over because at a healthcare capacity of $50,000 Canadian dollars ($CAD) for each additional fracture averted or for one QALY gained its probabilities of cost-effectiveness compared to the program promoting physical activity are 63% and 75%, respectively, which could be considered socially acceptable. Consideration of the indirect costs could change these findings.

## Introduction

Osteoporosis is a disease characterized by deterioration in the microarchitecture of bone tissue that leads to increased bone frailty and susceptibility to fragility fractures. In Canada and most Western countries, its prevalence in the population of postmenopausal women 50 to 54 years old is about 4.0%. This increases to 45% in women 85 to 89 years old.^1^ In women over 50 years old, bone loss leads to a lifetime risk of fractures of approximately 40%.^2^, ^3^ It has been estimated that two osteoporosis-related fractures occur every hour in women 50 years and older in Canada.^3^

Several interventions have been shown to be effective to prevent osteoporosis-related fractures. Primary prevention consists of interventions such as promotion of calcium and vitamin D supplements and of physical activity.^1^, ^4–6^ Screening aims at the identification of women at high risk followed by initiation of pharmacological therapy.^7^ Recently published 2010 osteoporosis best practice guidelines propose an integrated approach to osteoporosis management guided by an assessment of the patient's 10-year absolute risk of bone fractures.^4^ Pharmacological treatment is recommended for women who have at least 20% 10-year basal absolute risk.^4^ Yet in this framework, different intervention options exist.

To our knowledge, no Canadian study has compared the cost-effectiveness (CE) and cost-utility (CU) of those different options. Using a patient-level Markov model, we compared the expected CE and CU of 16 different interventions that covered three main scenario groups: (1) no national prevention program, which is the present situation in our jurisdiction; (2) a universal primary prevention program; and (3) a universal risk of fracture screening program.

## Materials and Methods

### Modeling and input parameters

**Figure 1:**
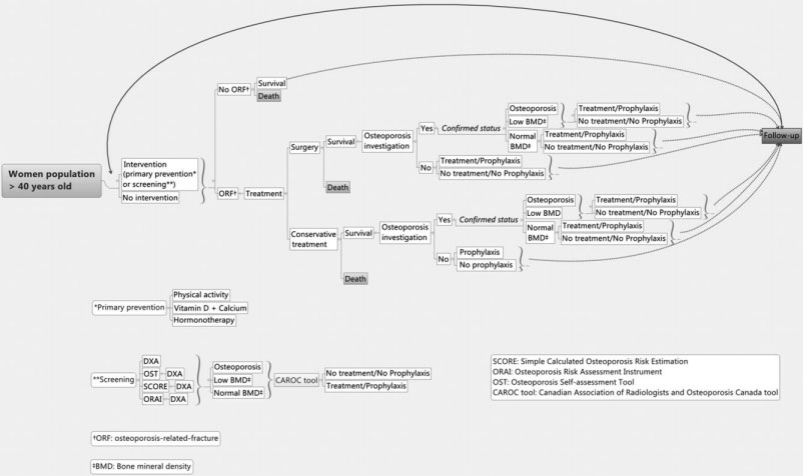
Decision model.

A patient-level Markov model (SPLMM) using an individual sampling approach^8^, ^9^ was used to simulate each of the 16 possible scenarios to compare (Fig. 1 and Table 2). The three most prevalent fracture sites were considered: hip, clinical vertebral, and wrist.^10–14^ The maximum number of hip fractures in a single individual was considered to be two in a lifetime because we assumed that all hip fractures lead to hemiarthroplasty. Because we considered age-specific annual probability of fracture by type of fracture and BMD, the state-transition model was divided into 1-year cycles.

A virtual population of 500,000 women 40 years old and older was generated. This population had the population age distribution of a typical industrialized country.^15^ The population was followed with 1-year cycles until all individuals had died. Detailed input parameters are presented in Table 1. Baseline parameters were retrieved from peer-reviewed published studies prioritized according to the following order: Quebec, other Provinces of Canada, United States, Europe, and Australia. A systematic search of the peer-reviewed literature, guidelines, and government reports was performed to define the range of values to be used for sensitivity analyses. Outcomes considered were the total number of fractures (wrist, hip, and clinical vertebral) for the entire population, as well as direct costs for the public healthcare system and quality-adjusted life-years (QALYs). Simulations were performed with two types of cohorts: (1) a single cohort of women 40 years old and over followed until their death; (2) the previous cohort to which were added annually a new cohort of 40-year-old women over the first 10 years of the simulation, as was performed in a previous work,^16^ which were also followed until their death.

**Table 1:** Input Parameters

Parameter	Base case	Range for sensitivity analyses	Distribution	References
BMD related-risk of fractures (hip, clinical vertebral, and wrist)	Calculated using data from references		–	2, 10, 14
Ten-year absolute risk of fractures and categorization (low-, moderate-, high-risk)	Calculated using data from references		–	17, 18
BMD distribution	Estimated from a representative cohort of 2104 women aged 40 years and older		–	18
Osteoporosis investigation after fracture	0.21	0.017–0.50	Uniform	19
Osteoporosis treatment/prevention after fracture	0.756 if osteoporosis; 0.294 if low BMD; 0.09 if normal BMD		–	19
Compliance rate to osteoporosis treatments	0.49	30–75	Uniform	20
RR death following hip and clinical vertebral fracture	4.31 (hip); 2.85 (vertebral); Probability of death = RRX probability of age-specific death probability in Quebec		–	15, 21
Acute rehabilitation for hip fracture	0.48		–	22
Long care (hip fracture)	0.20	0.10–0.282	Uniform	22–24
Wrist fracture surgery	0.18		–	25
Clinical vertebral fracture hospitalization	0.10		–	22
RR fracture				
Risedronate	Hip: 0.72; Clinical vertebral: 0.58; Wrist: 0.82	Hip: 0.58–0.88; Clinical vertebral: 0.50–0.67; Wrist: 0.74–0.90	Log normal	26
Vitamin D and calcium	0.88	0.83–0.95	Log normal	6
Physical activity (hip only)	0.62	0.54–0.69	Log normal	27
	Sensitivity/specificity	Sensitivity/specificity	Distribution	References
Performance of questionnaire								
OST	0.768/0.514	0.70–0.95/0.30–0.70	Uniform	28
SCORE	0.90/0.32	0.80–100/0.20–0.50	Uniform	29
ORAI	0.933/0.464	0.85–100/0.30–0.80	Uniform	30
Participation rate to interventions	0.531	0.30–0.70	Uniform	31
Utilities	Score calculated	Sensitivity analyses range	Distribution	References
Hip fracture								
Hospitalization postfracture	0.30	0.51–0.60	Uniform	32
Rehabilitation	0.56	0.63–0.70	Uniform	32
Postrehabilitation	0.85	0.73–0.90	Uniform	32
Clinical vertebral fracture								
Hospitalization	0.33		–	
Rehabilitation	0.68		–	
Postrehabilitation	0.85	0.76–0.90	Uniform	33, 34
Wrist fracture								
Emergency room	0.61		–	
Rehabilitation	0.88		–	
Postrehabilitation	1.00	0.82–1.00	Uniform	33
Item	Probability	Frequency	Cost ($CAD)/person	Distribution	References
Hip fracture					
Acute care (emergency and surgery)	1.00	1	4,070	–	22, 24, 35–37
Hospitalization	1.00	14 days	19,760 15,808–23,712	Uniform	38
Inpatient medical visits	1.00	14	229	–	35
Acute rehabilitation	0.48	30 days	24,639	–	22, 24, 35, 36
Long-term care	0.20	1 year	74,646	–	22, 24, 39
Follow-up	0.80	3 medical visits, 3 control X-rays, 7 physiotherapy sessions	990	–	22, 35–37
Clinical vertebral fracture					
Acute care (emergency visit)	1.00	1	1004	–	22, 35, 36
Hospitalization	0.1	9 days	8047 (6261–9891)	Uniform	22
Inpatient medical visits	1.00	9	146	–	22, 35
Follow up	1.00	2 control X-rays, 2 control medical visits, 7 physiotherapy sessions	550	–	22, 35, 36
Wrist fracture					
Acute care + conservative treatment	0.82	1	1250	–	22, 25, 35, 36
Acute care + surgery	0.18	1	3839	–	22, 25, 35, 36
Follow-up	1.0	2 control X-rays, 2 control medical visits, 7 physiotherapy sessions	467	–	22, 35, 36
Osteoporosis screening					
Medical visit and exams	–	1	160	–	–
DXA	–	1	107.5	–	1, 4, 35, 36, 40
Osteoporosis treatment					
Vitamin D and calcium	–	Annual	160	–	4, 41
Physical activity	–	Annual	5	–	4, 42
Biphosphonate (risedronate)	–	Annual	162.25	–	4, 41
Follow-up					
Follow-up medical visit	–	Annual	99.53	–	1, 35, 36, 43
Control DXA	–	2 years or 5 years (if low risk of fracture)	98.5	–	4, 35, 36
Discount rate			3% (0 and 5)	–	

BMD = bone mineral density; RR = relative risk; RRX = probability of death; OST = Osteoporosis Self-Assessment Tool; SCORE = Simple Calculated Osteoporosis Risk Estimation; ORAI = Osteoporosis Risk Assessment Instrument; $CAD = Canadian dollars; DXA = dual-energy X-ray absorptiometry.

The population was categorized in age groups of 5-year intervals (40–44 years, 45–49 years, etc.). However, the analyses showed that only a distinction between less than 65 years old and 65 and over brought specific age-related results. Only the results for these later groups are presented here.

### Options and scenarios

Table 2 presents the 16 options related to the three scenario groups that were compared.

**Table 2:** Simulated Options

Scenario group	Option/intervention	Description
No specific program	Status quo	Current situation where there is no specific universal primary prevention or universal screening
Universal primary prevention	Physical activity	Proposed for women who do not currently do physical activity; pharmacological treatment if a fracture occurs
	Vitamin D and calcium	Proposed for women who currently do not take vitamin D and calcium; pharmacological treatment if a fracture occurs
	Vitamin D and calcium + physical activity	Proposed for women who currently do not do physical activity and do not take vitamin D and calcium; pharmacological treatment if a fracture occurs
Universal screening	BMD/CAROC + physical activity	Universal screening by CAROC with BMD; pharmacological treatment for women with 10-year risk of fracture ≥20%; physical activity for women who do not need pharmacological treatment
	BMD/CAROC + vitamin D and calcium	Universal screening by CAROC with BMD; pharmacological treatment for women with 10-year risk of fracture ≥20%; vitamin D and calcium for women who do not need pharmacological treatment
BMD/CAROC+ vitamin D and calcium + physical activity	Universal screening by CAROC with BMD; pharmacological treatment for women with 10-year risk of fracture ≥20%; vitamin D and calcium + physical activity for women who do not need pharmacological treatment
	ORAI/CAROC + physical activity	Universal prescreening by ORAI tool; screening by CAROC with BMD for women who are positive according to ORAI; pharmacological treatment for women with 10-year risk of fracture ≥20%; physical activity for women who do not need pharmacological treatment
	ORAI/CAROC + vitamin D and calcium	Universal prescreening by ORAI tool; screening by CAROC with BMD for women who are positive according to ORAI; pharmacological treatment for women with 10-year risk of fracture ≥20%; vitamin D and calcium for women who do not need pharmacological treatment
	ORAI/CAROC + vitamin D and calcium + physical activity	Universal prescreening by ORAI tool; screening by CAROC with BMD for women who are positive according to ORAI; pharmacological treatment for women with 10-year risk of fracture ≥20%; vitamin D and calcium + physical activity for women who do not need pharmacological treatment
	OST/CAROC + physical activity	Universal prescreening by OST tool; screening by CAROC with BMD for women who are positive according to OST; pharmacological treatment for women with 10-year risk of fracture ≥20%; physical activity for women who do not need pharmacological treatment
	OST/CAROC+ vitamin D and calcium	Universal prescreening by OST tool; screening by CAROC with BMD for women who are positive according to OST; pharmacological treatment for women with 10-year risk of fracture ≥20%; vitamin D and calcium for women who do not need pharmacological treatment
	OST/CAROC + vitamin D and calcium + physical activity	Universal prescreening by OST tool; screening by CAROC with BMD for women who are positive according to OST; pharmacological treatment for women with 10-year risk of fracture ≥20%; vitamin D and calcium + physical activity for women who do not need pharmacological treatment
	SCORE/CAROC + physical activity	Universal prescreening by SCORE tool; screening by CAROC with BMD for women who are positive according to SCORE; pharmacological treatment for women with 10-year risk of fracture ≥20%; physical activity for women who do not need pharmacological treatment
	SCORE/CAROC + vitamin D and calcium	Universal prescreening by SCORE tool; screening by CAROC with BMD for women who are positive according to SCORE; pharmacological treatment for women with 10-year risk of fracture ≥20%; vitamin D and calcium for women who do not need pharmacological treatment
	SCORE/CAROC + vitamin D and calcium + physical activity	Universal prescreening by SCORE tool; screening by CAROC with BMD for women who are positive according to SCORE; pharmacological treatment for women with 10-year risk of fracture ≥20%; vitamin D and calcium + physical activity for women who do not need pharmacological treatment

BMD = bone mineral density; CAROC = Canadian Association of Radiologists and Osteoporosis Canada; ORAI = Osteoporosis Risk Assessment Instrument; OST = Osteoporosis Self-Assessment Tool; SCORE = Simple Calculated Osteoporosis Risk Estimation.

The first scenario, termed status quo, does not correspond to an absence of primary or secondary prevention, but to the absence of a specific national program to initiate preventive activities in women. In other words, this scenario considers the proportion of women who presently undertake preventive activities. Following a fracture, a woman may be investigated for osteoporosis or not.^19^ Depending on the investigation outcome, she has a certain probability of being treated with pharmacotherapy (risedronate) or of being proposed to take calcium and vitamin D.^19^, ^44^ In the baseline scenarios, risedronate is to be taken until death. However, in sensitivity analyses, we considered 5 years and 10 years duration of pharmacotherapy. The compliance rate to osteoporosis treatments in Canada was taken into account.^20^

The model considers the risk of death following a fracture^21^ and the proportion of women with a fracture who enroll in a physical rehabilitation program.^22^ It considers the specific effects of biphosphonate (risedronate), vitamin D + calcium and physical activity on the risk of hip, wrist, and clinical vertebral fractures by BMD and age category.^5^, ^27^ It also takes into account the probability for a woman with a wrist fracture to undergo surgery,^25^ with a hip fracture to be transferred to long-term care^22–24^ and ambulatory rehabilitation,^22^ and with a clinical vertebral fracture to be hospitalized.^22^

The second scenario refers to primary prevention of osteoporosis. We tested the options recommended by the 2010 Canadian guidelines on diagnosis and management of osteoporosis: (1) supplements of calcium and vitamin D; (2) promotion of physical activity (which can be simply walking every day); and (3) a combination of physical activity and calcium and vitamin D.^4^ The options were applied to the age-weighted proportion of women who, in the province of Quebec, do not practice some kind of physical activity according the definition of Statistics Canada,^18^, ^45^, ^46^ or do not take vitamin D and calcium supplements.^18^, ^46^ The baseline proportion of these women who adopt a preventive option was inferred from the participation rate in the Quebec national screening program for breast cancer.^31^ For the options that combine physical activity and supplementation of vitamin D and calcium, the simulation considered the highest effect of any of them on fracture risk reduction. When a fragility fracture occurred, the progression in the model was similar to the one described in the first scenario.

The third scenario refers to a universal screening program that would aim at identifying women at risk of having an osteoporosis-related fracture, using the Canadian Association of Radiologists and Osteoporosis Canada screening tool (CAROC), which is based on age, gender, bone mineral density, prior fracture, and prior use of glucocorticoids.^17^ This option complies with the 2010 Canadian guidelines on diagnosis and management of osteoporosis^4^ recommended by the Canadian Task Force on Preventive Health Care^1^ and the Canadian Consensus Conference on Osteoporosis 2006.^47^ The possibility for a prescreening step before considering women for BMD screening was also included in the simulation. The three questionnaires considered are those with the highest sensitivity/specificity related to being osteoporotic and/or that are validated for the Canadian population, namely: the Simple Calculated Osteoporosis Risk Estimation (SCORE)^29^; the Osteoporosis Risk Assessment Instrument (ORAI)^30^; and the Osteoporosis Self-Assessment Tool (OST).^28^ The baseline participation rate for the screening scenario was estimated to be the same as for primary prevention. The model took into account the tests' sensitivity and specificity. According to the prescreening and CAROC screening results, women are categorized into three groups: low risk (<10% 10-year risk of fracture), moderate risk (between 10% and 20% 10-year risk of fracture), and high risk (>20% 10-year risk of fracture) based on thresholds defined by the Canadian Association of Radiologists and Osteoporosis Canada.^17^ Low-risk patients receive a recommendation to adopt one of the preventive options (physical activity and/or vitamin D and calcium). A moderate risk implies preventive options or pharmacotherapy (risedronate) when other risk factors are present. A high risk implies pharmacologic treatment (risedronate). When a fragility fracture occurs, the progression in the model proceeds as described in the first scenario. The model considers that preventive or curative treatments are undertaken without interruption until death occurs.^4^

### Utilities

The Health Utilities Index III (HUI3) was used to score the utility of different health states that occurred in the model over time. These calculated utility scores were validated by an expert committee and were used in the base case scenarios. Published utilities as described in the literature^32–34^ were used in sensitivity analyses (Table 1).

### Costs

In Canada, all services considered as medically required (except ambulatory prescribed drugs) are generally provided exclusively within the public healthcare system and are free of charge. The Quebec Ministry of Health and the Public Medical Insurance perspectives were therefore considered. Only direct costs were estimated.

Cost items included fracture-related healthcare and rehabilitation services, long-term hospitalization for people with loss of autonomy following a fracture, prevention campaigns, primary screening for osteoporosis, drug prophylaxis and treatment of osteoporosis, and medical follow-up of patients with and without osteoporotic fracture. Cost of ambulatory-provided drugs was attributed to the public healthcare system and not distributed between patients and public insurers because of the complexity of coverage eligibility in the province.

The fiscal year 2007–2008 was used to calculate unit prices presented in Table 1. Unit prices for services obtained in the public health care were provincial averages calculated from the Quebec government databases (Système d'information financière et opérationnelle [SIFO] and All Patient Refined–Diagnosis Related Groups [APR-DRG]). Unit prices of clinical activity centers were increased to reflect support activities centers using the direct method.^48^ Costs for laboratory and imaging tests were based on the technical units in the province of Quebec.^36^ The average cost of national campaigns of prevention in Quebec ($3–5 Canadian dollars [$CAD] per capita) was used as the cost for a physical activity promotion campaign.^42^ Public health insurance fees paid to general practitioners and specialists were considered.^35^ For pharmaceuticals, the cheapest in the list of drugs covered by the public health insurance was used (eg, risedronate as the biphosphonate), to which was added a 6% for wholesalers and the pharmacist's prescribing fee paid by the public insurance. The average per diem calculated by the Ministry of Health and Social Services was used for long-term hospitalization.^39^ All costs and outcomes were discounted at a rate of 3%, and sensitivity analyses were performed with values of 0% and 5%.

### Simulations

In order to produce a distribution curve, simulations for each option were repeated 1000 times, each time on a newly generated (ie, different) virtual population. Simulations were performed with SCHNAPS,^8^, ^9^ a simulator running on the COLOSSE supercomputer of the CLUMEQ consortium (www.clumeq.ca).

### Sensitivity analyses

One-way sensitivity analyses were performed using the variables considered most influent on the outcomes in order to evaluate the eventual impact of each single parameter on the results. We tested the minimum and the maximum value for each of these variables (Table 1). Subsequently, multiway probabilistic sensitivity analyses were performed. Simulation for each option was also repeated 1000 times to ensure the stability of results. A CE and a CU acceptability curve were produced in order to better define the uncertainty of the incremental cost-effectiveness ratios (ICERs) of the best alternatives.^49^

### Model validation

The model and simulation data were validated by three osteoporosis experts. Data produced were validated by comparison with expected data (such as the number of fractures, mortality rates per age, and costs and effectiveness of interventions).^14^ A less than 5% difference with expected results based on the literature was sought. For example, our model estimated the proportion of 40-year-old women who would have a fracture during their remaining lifetime to be 17.9% for hip fractures and 16.07% for wrist fractures, and 15.83% for clinical vertebral, which were similar to published estimates.^2^, ^13^, ^14^

### Ethics committee

This project was approved by the Research Ethics Committee of Laval University. None of the authors felt that he/she was in conflict of interest while participating to this study.

## Results

Results are presented for women 40 to 64 years old and for women 65 years old and over at the start of the simulation. Results for other age categories (40–49, 50–59, 60–69, 70–79, and 80+ years) are available upon request.

The most effective option for reducing the total number of fractures appeared to be a universal BMD testing program followed by the estimation of the 10-year absolute fracture risk with the CAROC tool, and the treatment of women at high risk for osteoporosis-related fractures and the promotion of physical activity, as well as the intake of vitamin D and calcium among non–high-risk women.

However, in terms of CE (Table 3), for women 40 to 64 years old at the beginning of the simulation, a program promoting physical activity for sedentary women emerged as the most interesting option. It is effective and cost-saving. Compared to the status quo, it is dominant. Scenarios based on screening for women at risk for fracture, then treating those considered at high risk and promoting preventive activities for the others are effective but their ICERs compared to the cheapest alternative are all larger than $100,000 ($CAD) per fracture averted.

**Table 3:** Cost-Effectiveness Results

Option	Total costs ($CAD)	Incremental costs	Total fractures	Fractures averted	ICERs
Women 40–64 years old (*n* = 363042)					
Physical activity	1,752,926,600		215,330		Baseline^a^
Status quo	1,755,241,287	2,314,687	219,013	−3683	–
OST/CAROC + physical activity	2,005,406,312	250,165,025	213,940	5073	–
ORAI/CAROC + physical activity	2,009,581,197	474,885	213,925	15	–
SCORE/CAROC + physical activity	2,011,844,082	2,262,885	213,930	−5	–
BMD/CAROC+ physical activity	2,016,897,393	5,053,311	213,890	40	–
OST/CAROC + vitamin D and calcium	2,085,851,423	68,954,030	213,826	64	–
OST/CAROC +physical activity + Vitamin D and calcium	2,096,519,944	668,521	213,824	2	–
ORAI/CAROC + vitamin D and calcium	2,097,619,345	1,099,401	213,825	−1	–
SCORE/CAROC + vitamin D and calcium	2,097,676,214	56,869	213,820	5	–
BMD/CAROC + vitamin D and calcium	2,105,354,023	7,677,809	213,834	−14	–
SCORE/CAROC + physical activity + vitamin D and calcium	2,107,272,843	1,918,820	211,976	1858	105,649
ORAI/CAROC + physical activity + vitamin D and calcium	2,107,327,214	54,371	211,990	−14	–
BMD/CAROC + physical activity + vitamin D and calcium	2,115,595,462	8,268,248	211,952	38	346,776
Physical activity + vitamin D and calcium	214,2763,906	27,168,444	212,180	−228	–
Vitamin D and calcium	2,144,102,484	13,385,578	215,131	−2951	–
Women ≥65 years old (*n* = 136958)					
Physical activity	1,002,395,979		61,976		
Status quo	1,025,394,048	22,998,069	63,564	−1588	–
CAROC + physical activity + vitamin D and calcium	1,071,691,507	46,297,459	60,825	2739	60,205
OST/CAROC + physical activity + vitamin D and calcium	1,086,269,626	14,578,119	61,280	−455	–
SCORE/CAROC + physical activity + vitamin D and calcium	1,089,941,050	3,671,424	61,219	61	–
ORAI/CAROC + physical activity + vitamin D and calcium	1,091,247,887	1,306,837	61,210	9	–
Physical activity + vitamin D and calcium	1,092,852,516	1,604,629	61,187	23	–
OST/CAROC + vitamin D and calcium	1,104,577,805	11,725,289	62,073	−86	–
Vitamin D and calcium	1,107,165,714	2,587,909	62,215	−142	–
SCORE/CAROC + vitamin D and calcium	1,109,593,435	2,427,721	62,057	158	–
ORAI/CAROC + vitamin D and calcium	1,110,539,440	946,005	62,073	−16	–
CAROC + vitamin D and calcium	1,111,676,305	1,136,865	61,999	74	–
OST/CAROC + physical activity	1,121,427,790	9,751,485	62,024	−25	–
ORAI/CAROC + physical activity	1,121,744,178	316,388	61,904	120	–
SCORE/CAROC + physical activity	1,121,755,853	11,675	61,922	−18	–
CAROC + physical activity	1,122,808,961	1,053,108	61,901	21	–

$CAD = Canadian dollars; ICER = incremental cost-effectiveness ratio; OST = Osteoporosis Self-Assessment Tool; CAROC = Canadian Association of Radiologists and Osteoporosis Canada; ORAI = Osteoporosis Risk Assessment Instrument; SCORE = Simple Calculated Osteoporosis Risk Estimation; BMD = bone mineral density.

a Less expensive strategy.

However, in women 65 years old and over, a BMD screening program followed by estimation of the 10-year absolute risk of fracture using the CAROC tool and pharmacological treatment for women considered at high risk for fractures, whereas promoting of preventive activities for others could be considered as a reasonable alternative, because its ICER compared to a program aiming at increasing physical activity among women is less than $65,000 ($CAD) per fracture avoided.

From a CU perspective, results are similar (Table 4). For women who younger than 65 years old, a program to incite sedentary women to undertake physical activities emerged also as the less costly, the more effective and the one with the most interesting CU ratio. A BMD screening program with estimation of the 10-year absolute risk of fracture using the CAROC tool and pharmacological treatment for women considered at high risk for fractures, as well as the promotion of preventive activities for others also emerges as a possible alternative because its ICER compared to a program promoting physical activity is about $50,000 ($CAD) per QALY gained.

**Table 4:** Cost-Utility Results

Option	Cost/person ($CAD)	Incremental cost/person	QALYs/person	Incremental QALYs	ICURs
Women 40–64 years old (*n* = 363042)					
Physical activity	4,828		20.7225	Baseline^a^	
Status quo	4,835	7	20.71274	−0.00976	–b
OST/CAROC + physical activity	5,524	689	20.72022	0.007446	–
ORAI/CAROC + physical activity	5,535	9	20.72273	0.00251	–
SCORE/CAROC + physical activity	5,542	7	20.723	0.00027	–
BMD/CAROC + physical activity	5,556	14	20.72282	−0.00018	–
OST/CAROC + vitamin D and calcium	5,746	204	20.72308	0.00026	–
OST/CAROC + physical activity + vitamin D and calcium	5,775	29	20.72611	0.00303	–
ORAI/CAROC + vitamin D and calcium	5,778	3	20.72097	−0.00514	–
SCORE/CAROC + vitamin D and calcium	5,780	2	20.72144	−0.00047	–
BMD/CAROC + vitamin D and calcium	5,799	19	20.72081	−0.0063	–
SCORE/CAROC + physical activity + vitamin D and calcium	5,804	5	20.72655	0.00574	–
ORAI/CAROC + physical activity + vitamin D and calcium	5,805	1	20.72584	−0.00071	–
BMD/CAROC + physical activity + vitamin D and calcium	5,827	22	20.72672	0.00088	239,573
Physical activity + vitamin D and calcium	5,902	75	20.72576	−0.00086	–
Vitamin D and calcium	5,906	4	20.71946	−0,0064	–
Women ≥65 years old (*n* = 136958)					
Physical activity	7,319		11.31492		
Status quo	7,487	168	11.29549	−0.01943	
BMD/CAROC + physical activity + vitamin D and calcium	7,825	338	11.32407	0,02858	55,300
OST/CAROC + physical activity + vitamin D and calcium	7,931	106	11.31566	−0.00841	–
SCORE/CAROC + physical activity + vitamin D and calcium	7,958	27	11.31702	0.00136	–
ORAI/CAROC + physical activity + vitamin D and calcium	7,967	9	11.31813	0.00111	–
Physical activity + vitamin D and calcium	7,979	12	11.3193	0.00117	–
OST/CAROC + vitamin D and calcium	8,065	86	11.30823	−0.01107	–
Vitamin D and calcium	8,084	19	11.30893	0.0.0007	–
SCORE/CAROC + vitamin D and calcium	8,102	18	11.30711	−0.00182	–
ORAI/CAROC + vitamin D and calcium	8,108	6	11.31093	0.00382	–
BMD/CAROC + vitamin D and calcium	8,117	9	11.30714	−0.00379	–
OST/CAROC + physical activity	8,188	71	11.31116	0.00402	–
ORAI/CAROC + physical activity	8,190	2	11.31085	−0.00031	–
SCORE/CAROC + physical activity	8,191	1	11.31238	0.00153	–
BMD/CAROC + physical activity	8,198	7	11.31195	−0.00043	–

$CAD = Canadian dollars; QALY = quality-adjusted life-year; ICUR = incremental cost-utility ratio; OST = Osteoporosis Self-Assessment Tool; CAROC = Canadian Association of Radiologists and Osteoporosis Canada; ORAI = Osteoporosis Risk Assessment Instrument; SCORE = Simple Calculated Osteoporosis Risk Estimation; BMD = bone mineral density.

a Less expensive strategy.

b Dominated strategies are those that were found to be less efficacious and more expensive than another strategy (strict dominance) or to have an incremental cost-effectiveness ratio that is greater than that of the next, more effective, and more expensive alternative (extended dominance).

The addition of new cohorts of 40-year-old women for the first 10 years of the simulation did not influence the ranking of the most desirable options and even improved their overall CU and CE (data not shown).

### Sensitivity analyses

CE and CU results were robust to sensitivity analyses: the ranking of the most promising scenarios remained unchanged. However, we observed that a change of certain parameters did have a sensible impact on CE of interventions when compared to the base case scenario. For example, with a stronger participation rate to prevention or screening strategies, the ICER of CAROC + vitamin D and calcium + physical activity was improved; ie, it was 17% lower. The same options improved by 25% in the case of a higher efficacy of risedronate. In contrast, lower effects of vitamin D and calcium as well as physical activity, a higher discount rate (5%), and a lower participation rate increased the ICERs compared to the base case scenario but did not change the ranking of the most promising options. In multiway sensitivity analyses, results were also robust. The rank order of the strategies did not change and the ICER for each strategy remained relatively stable (data not shown).

**Figure 2:**
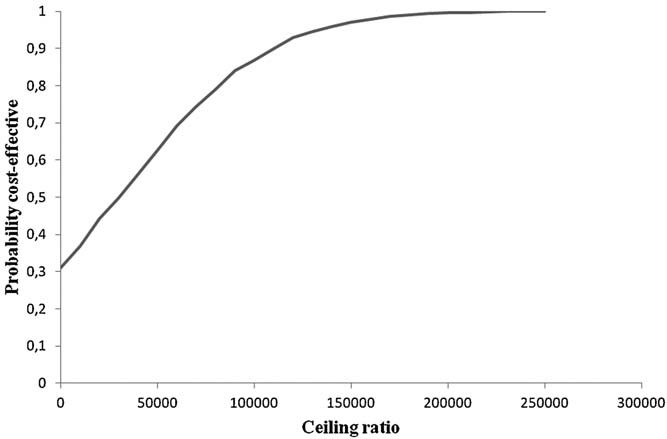
Cost/effectiveness acceptability curve BMD/CAROC + vitamin D and calcium + physical activity versus physical activity for women ≥65 years old.

**Figure 3:**
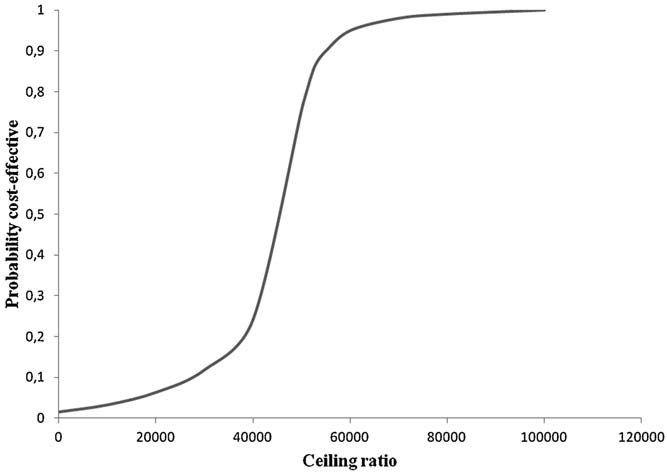
Cost/utility acceptability curve BMD/CAROC + vitamin D and calcium + physical activity versus physical activity for women ≥65 years old.

When CE and CU acceptability curves were produced for women 65 years old and over to compare the program promoting physical activity with a BMD screening program with the supplementation of vitamin D, calcium, and promotion of physical activity suggested to women at low and middle risk, it appeared that at a ceiling ratio of $50,000 ($CAD) generally suggested as a threshold to adopt an intervention,^50^ there is respectively a probability of 63% and of 75% that the screening program is CE (Figs. .2 and 3).

## Discussion

This work presents data on CE and CU of 16 different options for the prevention of osteoporosis-related fractures including those proposed by the recent 2010 Canadian guidelines on diagnosis and management of osteoporosis. To our knowledge, our study is the first modeling approach that compared prevention, screening, and the use of the CAROC tool for the identification of women who should benefit from a pharmacological or a preventive intervention. Other modeling approaches have generally used the BMD *T*-score as a criterion for pharmacological treatment.

In terms of effectiveness (fractures averted), the preferred option was BMD screening for women for osteoporosis-related fracture and the determination of their 10-year absolute risk with the CAROC tool, followed by a pharmacologic treatment for those at risk and a nonpharmacologic preventive intervention (physical activity plus vitamin D and calcium) for those at moderate and low risk. However, due to its lower costs, the promotion of physical activity (followed by treatment when a fracture occurs) is the most CE option for women between 40 and 64 years old. Indeed, because all options have a modest effect on reducing the number of fractures in the general population compared to the changes in costs, effectiveness does not significantly influence CE ratios. This is particularly obvious for women younger than 65 years old at the beginning of the simulation and could be due to the fact that the prevalence of osteoporosis and osteoporosis-related fractures is lower in this group. For older women at the beginning of the simulation, a BMD screening program might be considered as the best CE option. Its ICER compared to the promotion of physical activity is in the order of $60,000 ($CAD) per fracture averted and $50,000 ($CAD) per QALY gained. The probabilistic sensitivity analyses showed that at $50,000 ($CAD) per additional fracture averted, the probability that this option is CE is 63%. At $50,000 ($CAD) per QALY gained, it is 75%. A ceiling ratio of $50,000 ($CAD) is generally suggested as a threshold to adopt an intervention in North America.^50^ One notes that a BMD screening program for women 65 years old and over is coherent with the Canadian^4^ and National Osteoporosis Foundation (NOF)^51^ guidelines.

Ranking of the various options tested by CU and CE appeared similar. However, differences in QALYs were marginal, and might be explained by the fact that life expectancy differs very little from one option to another, and that the impact of events on utilities of a few individuals inside the virtual population does not much influence the average utility of the entire population. Similar results were reported in other simulations.^52^, ^53^

This research has also some limitations. The main limitations of such a study are related to the mapping of the complex reality. Indeed, some degree of simplification was needed.^54^, ^55^ For example, our model considered only three sites of osteoporosis-related fracture (hip, clinical vertebral, and wrist), in spite of the fact that osteoporosis-related fractures might affect other sites such as the proximal humerus and the pelvis. Taking these other sites into account could increase the costs of strategies and affect the CE and/or CU ratios. In addition to that, we did not take into account nonclinical vertebral fractures as we considered that women with these kind of fractures do not often seek medical help because the majority of them do not have back pain or other symptoms, thus do not impact costs very much. We acknowledge that these fractures may cause some disutility to patients that might affect QALYs results.

Another limitation to the present study relates to the rate of participation in interventions to prevent osteoporosis-related fractures. We used the same participation rate as the rate of the Quebec public breast cancer screening program. Yet reality might be slightly different because osteoporosis and breast cancer are different problems. We considered the Quebec breast cancer screening program participation rate because it is the only universal screening program in our population that targets women and for which data exist. Furthermore, we assumed that the participation rates are similar for all interventions (screening and lifestyle), which may not reflect the reality because we know that, in general, the uptake rates related to behavior changes are low when compared to screening with noninvasive tests.^56^, ^57^ Another limitation of this work is that we did not model the side effects of drug treatments or the potential additional benefits of osteoporosis prevention and treatment on other health problems (eg, the effects of physical activity on cardiovascular problems). Also, patients were considered as compliant or not, and the model did not consider the reduced effects of poor observance. In addition, the model assumed the same adherence rate for pharmacological therapy as for lifestyle changes. This might not reflect the real world where lifestyles are difficult to change.^56^, ^57^ However, we believe that the probabilistic sensitivity analyses done have solved partially this issue. Another limit is that the model did not consider the cumulative effect of various interventions performed concomitantly, such as physical exercise and vitamin D and calcium intake. Indeed, there is no data available on the combined effect of these interventions.^4^ Adopting a conservative approach, we considered the highest effect of any of them on fracture risk reduction, knowing that this might not adequately reflect reality, because a combination effect could increase the effectiveness of some interventions. Also, our analyses were limited to direct costs borne by the public healthcare perspective. The fact that we did not take account of indirect costs could provide another ranking, especially for physical activity programs for which indirect costs are high. For example, we did not consider investments by the government in sports facilities or individual direct costs spent by individuals to use these facilities.

Finally, one should be cautious about generalizing our results even though the scenarios were chosen on the basis of reasonable practices promoted for the entire Canadian context.^48^, ^54^ Regarding other countries, one might suppose that our results could be reproduced in other healthcare systems because the CAROC-based screening tool recommended in Canada has a 90% concordance in risk assessment with the FRAX tool preferred in other countries such as the UK, United States, Sweden, and Switzerland.^17^, ^19^, ^58^ In any case, whether these results apply to other healthcare jurisdictions remains to be confirmed.

## Conclusion

A program promoting physical activity is the most CE and CU option for women of 40 to 64 years. A BMD screening and treatment based on 10-year absolute risk of fracture calculated by the CAROC tool can be considered as a reasonable alternative for women who are 65 years old or more, if an incremental cost of $50,000 ($CAD) per additional fracture averted with a probability of CE of 63%, and $50,000 ($CAD) per QALY gained with a probability of CE of 75% are considered as socially acceptable.

## Disclosures

All authors state that they have no conflicts of interest.
